# Association of Screen Content With Early Development Among Preschoolers in Shanghai: 7-Day Monitoring Study With Auto Intelligent Technology

**DOI:** 10.2196/65343

**Published:** 2025-03-05

**Authors:** Hao Chen, Yi Sun, Sha Luo, Yingyan Ma, Chenshu Li, Yingcheng Xiao, Yimeng Zhang, Senlin Lin, Yingnan Jia

**Affiliations:** 1 Preventive Medicine and Health Education Department School of Public Health Fudan University Shanghai China; 2 Xuhui Maternity and Child Healthcare Center Shanghai China; 3 Shanghai Eye Hospital Shanghai Eye Diseases Prevention & Treatment Center Shanghai China; 4 Department of Ophthalmology School of Medicine Shanghai Jiao Tong University Shanghai China

**Keywords:** types of screen content, screen time, intelligent technology, early development, preschool

## Abstract

**Background:**

It is unclear how exposure to different types of screen content is associated with early development among preschool children.

**Objective:**

This study aims to precisely evaluate the screen exposure time across different content types and to explore the associations with the Ages and Stages Questionnaire, Third Edition (ASQ-3) score and 5 capacity domains in children aged 34.5-66 months.

**Methods:**

This monitoring study used intelligent technology to collect data on the 7-day screen time and the time spent viewing each content type. The participants were 2 groups of Shanghai kindergarten kids. The data were collected between March 2023 and July 2023. Screen exposure data (total daily time and time for each type of content) were collected from children aged between 34.5 and 66 months. A self-designed questionnaire and the Healthy Screen Viewing for Children intelligent technology app were used to assess screen exposure to all media and tablets. The ASQ-3 was used to assess early development in children aged 34.5-66 months.

**Results:**

In the 535-child sample, the results of linear regression analysis indicated that both screen time of more than 60 minutes and exposure to smartphones and tablets were negatively associated with ASQ-3 score. Among 365 participants with data collected by the Healthy Screen Viewing for Children app, median regression showed that the median total ASQ-3 score was negatively associated with screen time for noneducational content (β=–.055; 95% CI –0.148 to –0.006; *P*=.03), screen time for both educational and noneducational content (β=–.042; 95% CI –0.081 to –0.007; *P*=.001), and fast-paced content (β=–.034; 95% CI –0.062 to –0.011; *P*=.049). The median gross motor score was negatively associated with screen time for parental guidance-13–rated content (β=–.015; 95% CI –0.022 to 0.009; *P*=.03), educational and noneducational content (β=–.018, 95% CI –0.038 to –0.001; *P*=.02), static content (β=–.022; 95% CI –0.050 to 0.007; *P*=.02). This study also revealed that the median fine motor score was negatively associated with screen time for guidance–rated content (β=–.032, 95% CI –0.057 to –0.003; *P*=.006), parental guidance (PG) rated content (β=–.020; 95% CI –0.036 to –0.007; *P*=.004), noneducational content (β=–.026; 95% CI –0.067 to –0.003; *P*=.01), both educational and noneducational content (β=–.020; 95% CI –0.034 to –0.001; *P*<.001), fast-paced content (β=–.022; 95% CI –0.033 to –0.014; *P*<.001), static content (β=–.034; 95% CI –0.050 to 0.018; *P*<.001), animated content (β=–.038; 95% CI –0.069 to –0.001; *P*=.004), and screen use during the daytime (β=–.026; 95% CI –0.043 to 0.005; *P*=.005).

**Conclusions:**

The results indicated that the time spent viewing noneducational, static, fast-paced, and animated content was negatively associated with early development among preschool children. Limiting screen time in relevant aspects is recommended.

## Introduction

Since iPads and similar touch-enabled media technological devices are becoming increasingly popular worldwide, the increasingly younger trend of screen exposure may threaten the early development of toddlers and preschool children [1]. Although the World Health Organization [2] and pediatric societies worldwide (eg, Australian [3] and Canadian [4] 24-Hour Movement Guidelines) have recommended no more than 1-2 hours per day for children aged 2 to 5 years, the global prevalence of children who meet the screen time guidelines (1 hour/day) is only 35.6% (95% CI 30.6%-40.9%) [5]. Excessive screen time is associated with child language skills [6]; development [7]; mental health symptoms, including irritability, inattention, and hyperactivity [8]; obesity [9,10]; and physical inactivity [11,12].

The most active and most vulnerable period for brain and neural development is from the ages of 2 to 5 years [13] because children are able to generalize an action learned based on 2D screen images to a real 3D situation beginning at 2 years of age [14]. In addition to excessive screen time, there is still controversy about whether and how exposure to screen content during early life could influence children’s physical, psychological, and social development. Previous studies have shown that early exposure to violent entertainment programs is associated with aggressive behavior in childhood [13], and watching entertainment programs has adverse effects on cognitive development [15]; moreover, a cohort study showed that viewing entertainment programs was associated with greater expressive word skills [16]. Several intervention experiments have shown that watching cartoons with a fast screen-switching rate reduces executive function in children, but exposure to content with a slow screen-switching rate has no significant effect on executive function in children [17,18]. Several studies have suggested that playing games can improve children’s cognitive abilities, such as attention, visual processing, and spatial imagination, in the short term [19], but the long-term learning and educational benefits are not obvious [20].

In general, there is an urgent need for greater awareness about the complexities of the impact of exposure to screen content on children’s development, especially regarding touch-enabled media technology [21]. The most recent umbrella review of screen time studies, also calls for more objective measurements [22]. However, previous research seems simple and unable to match the pace of technological advances because the majority of screen exposure measurements are based on parent-reported screen viewing time and content, which might be susceptible to recall bias [1,23]. Conversely, the World Health Organization recommends that toddlers view high-quality content but fails to specify what defines such quality or offers representative examples [2]. Therefore, the primary aim of this study was to collect real-time screen exposure data through intelligent monitoring technology and then examine the association between screen viewing content and time and children’s early development. These results may be beneficial for defining high-quality evidence for direct future recommendations.

## Methods

### Study Design and Population

This monitoring study was conducted in 2 districts of Shanghai, Xuhui District (urban), and Pudong New Area (suburban), from March 2023 to July 2023. A random sampling method was used to select a kindergarten from each of the 2 districts. For each kindergarten, a cluster sampling method was used to select 5 classes, in which the average number of children in each class was 30, from the junior, middle, and senior grades. A total of 535 preschool children were included in this study, and they were required to use the Huawei iPad for 1 week. During the recruitment process, we repeatedly emphasized that children should use tablets according to their daily habits at home and that parents should neither indulge in children’s use which may lead to an overestimation of screen exposure duration nor overly regulate it which may result in an underestimation of screen exposure duration. All the children were provided with intelligent monitoring technology and completed a developmental screening questionnaire. The minimum sample size was calculated to be 462 by using the following formula: 
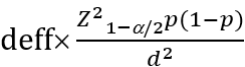
, where the reported prevalence of abnormal development among nationwide Chinese preschool children was approximately 10.8% to 26.2% [24]. In this study, the prevalence (p) was 10.8%, the type I error (α) was .05, the *z*_1-/2_ was 1.96, the precision (d) was 0.04, and the design effect (deff) was 2 [25]. The inclusion criteria for participant enrollment were as follows: (1) children aged between 2 and 6 years; (2) children whose parents or other caregivers allowed them to use screen devices, such as televisions, smartphones, iPads, and computers; and (3) children whose parents or other caregivers understood the questionnaire.

### Measures

#### Screen Exposure Monitoring Technology

The “Healthy Screen Viewing for Children (HSVC)” mobile app, developed by the Department of Preresearch of Huawei Corporations and installed on the Huawei iPad, was used to automatically collect data on screen time and screen content while running in the background. The screen time was calculated by obtaining the start time point and end time point as well as the sum of the duration of use for every app. Correspondingly, app name labels, namely, screen content information, were also collected simultaneously (the specific data collection and storage procedures are shown in Figure S1 in Multimedia Appendix 1). Considering that video applications (eg, YouTube and Netflix) cannot reflect the viewing program according to the application name, the HSVC application takes screenshots every 60 seconds to obtain images of the screen as an important source of data on-screen content. All the abovementioned screen exposure data were uploaded in real-time to the cloud server of Huawei Corp and can be downloaded from the Huawei Research Platform. Based on the data of 268 preschool children from the accuracy survey, the accuracy between the screen-time category from the HSVC records and the 24-hour diary according to the Bayesian classification of the testing set and training set were 0.96 and 0.93, respectively; moreover, the κ value of representativeness was 0.61.

#### Classification of Screen Content

The 7-day monitoring study ultimately collected 138.98 thousand screen records and 41,812 screenshot images. Based on previous studies and the Delphi method, screen content type was classified into the following six regulations.

- App function direction: entertainment videos, video games, tools, educational videos, and short videos.
- Suggested audience: General audience, parental guidance suggested (PG), parents strongly cautioned (PG-13), and restricted according to the film rating system formulated by the Motion Picture Association of America [26].
- Educational direction: Educational and noneducational content.
- Screen pace direction: The pace of content was defined as 2 episodes viewed for the number of times a complete change occurred (eg, from the living room to the stadium) according to a previous standard [18], and we categorized pace into fast-paced, medium-paced, and slow-paced content (switched more than 6 times per minute, 2 to 6 times per minute, and less than 2 times per minute, respectively).
- Screen interaction direction: Static, touch-enabled, and interactive for children and caregivers.
- Realistic or animated direction: The content corresponded to the program or game, which mainly included realistic content, animated content, and both realistic and animated content.

The specific classification procedures and standards are shown in Table S1 in Multimedia Appendix 1.

#### Developmental Screening

The Chinese version of the Ages and Stages Questionnaire, Third Edition (ASQ-3), which is a widely used, parent-reported screening tool, was used for developmental screening. The ASQ-3 comprises the following 5 domains and includes 30 items: communication (6 items), gross motor (6 items), fine motor (6 items), problem-solving (6 items), and personal-social (6 items) [27]. Each item is scored on a 3-point scale ranging from 10=yes to 5=sometimes to 0=not yet. The total ASQ-3 score is obtained by summing the corresponding item scores, with a higher ASQ-3 score indicating a preferable development level [28,29]. After validation via standardized testing of developmental (Bayley Scales of Infant Development) and intellectual (Stanford-Binet Intelligence Test–4th Edition) skills, the ASQ-3 was found to have moderate to high sensitivity (0.70-0.90) and specificity (0.76-0.91), as well as strong concurrent validity (*r*=0.85) and internal consistency (α=.51-.87) [30,31]. The ASQ-3 was released web-based through the Wenjuanxing platform. It included the filling instructions for ASQ-3. Parents were required to conduct evaluations on children in a quiet environment and then fill in the questionnaires themselves. We also provided contact information so that parents could give feedback in a timely manner and solve problems when encountering difficulties in filling out the questionnaires.

#### Covariates

Based on the results of univariate analysis of demographic characteristics, lifestyles, Ages and Stages Questionnaire (ASQ) scores, and each dimension (Table S2 in Multimedia Appendix 1), we respectively selected the indicators that had statistically significant associations with ASQ and its 5 dimensions as covariates and incorporated them into the regression models of ASQ and each dimension respectively, in order to evaluate the associations between screen exposure content and ASQ more accurately. In addition, we investigated children’s screen exposure in kindergartens. The results of 70 questionnaires showed that the screen exposure in schools was background screen exposure (such as playing videos and music in the classroom during recess gymnastics time while children were doing sports outdoors). The median duration of background screen exposure was P_50_=30 (20-30) minutes, and children rarely actively obtained information from background exposure. Therefore, this study did not include background screen exposure as a covariate in the analysis.

#### Caregiver-Reported Questionnaire

Based on the literature, the covariates in this study included child age, child gender, caregiver, maternal educational attainment, paternal educational attainment, monthly income, single child status, and health-related behaviors, including day sleep time, night sleep time, indoor physical activity level, outdoor physical activity level and screen control by caregivers.

A screen exposure questionnaire was used to measure screen time on all media to compensate for the inability of the HSVC to collect data from television, computers, and other nontablet media. The screen times for televisions, personal computers, smartphones, and tablets were derived from graded responses (0 minute, 1-29 minutes, 30-59 minutes, 60-120 minutes, or >120 minutes) to the question “How long does your child spend using TVs/PCs/cell phones/tablets on an average day?” The age at first screen exposure was derived from frequency responses (under the age of 1 year, 1-2 years, 2-3 years, or older than 3 years). The types of on-screen content were derived from ranked responses (eg, mainly educational or mainly entertainment or both educational and entertainment) to the question “What types of on-screen content is your child exposed to?” The response methods used for the type of on-screen content, namely, age restriction, animated direction, fast-paced, and touch-enabled, were similar to those used for educational direction. Caregivers’ interactions with children when viewing programs and playing games are related to early development; thus, the use of co-viewing programs and co-playing games was derived from frequency responses, which included seldom, sometimes, and often.

### Statistical Analysis

Participant characteristics are presented as frequencies with percentages and as the means with SDs. First, we used descriptive analysis to show the demographic characteristics of the participants in the whole sample with questionnaire data and in the subsample with HSVC device records. Second, a 2-tailed Student *t* test and ANOVA were performed to explore the distribution of the total ASQ-3 score for the whole sample and the scores on the 5 domains according to demographic characteristics, lifestyle, screen time category of the 4 media types, screen use styles, and type of screen exposure content, as shown in Tables S2 and S3 in Multimedia Appendix 1. Then, 6 multiple linear regression analyses were performed to examine the associations between the total ASQ score and the scores on the 5 domains (dependent variable), screen time category of the 4 media types, screen use styles, and type of screen exposure content (independent variables) adjusted for age, gender, parental educational attainment, and monthly income (control variable; Table S4 in Multimedia Appendix 1). Furthermore, Spearman correlation analysis was performed to examine the univariate association between the total ASQ score and the scores on the 5 domains and between screen viewing time and exposure to different types of content according to the HSVC application, as shown in Table S5 in Multimedia Appendix 1. Finally, median regressions were used to examine the nonlinear correlation trend between the median score on the ASQ and median time of exposure to content according to the HSVC sample application, as shown in Table S6 in Multimedia Appendix 1. The estimates of the independent variables for the total ASQ score and the domain scores were summarized using regression coefficients (β) and 95% CIs. Statistical analyses were performed using R software (version 4.3.2; R Foundation for Statistical Computing).

### Ethical Considerations

Before the participants underwent screen exposure monitoring and questionnaire surveys, we informed their parents (legal guardians) of the whole process of participation in the surveys as well as the benefits and risks involved, and the legal guardians signed the informed consent. This study conformed to the ethical guidelines of the 1975 Declaration of Helsinki and was approved by the Ethics Committee for Medical Research at the School of Public Health, Fudan University (IRB#2023-11-1088). When extracting data from the Huawei Research Platform and the web-based questionnaire library for analysis, all the data were deidentified, and an anonymous study ID was used as an identifier for each participant. In this study, we provided a gift of a paintbrush set, worth approximately US $15, to each child participating in the research.

## Results

### Participant Characteristics

As shown in Table 1, among a total of 535 children and 365 preschool children with tablet use, 298 (55.7%) and 205 (56.2%) were boys, and more than half of the participants’ caregivers were their mothers (n=297, 55.5% and n=205, 55.6%, respectively). The majority of participants’ parents had a bachelor degree (mothers: n=290, 54.2% and n=196, 53.7%; fathers: n=286, 53.5% and n=194, 53.2%). Most of the participants’ monthly incomes were over US$ 1750 (n=309, 57.8% and n=202, 55.4%). Nearly half of the participants were only children (n=272, 50.8% and n=185, 50.7%). Only 50 (9.3%) and 33 (9.1%) of participants had insufficient sleep during the day, while 412 (77%) and 280 (76.7%) of participants had insufficient sleep during the night (less than 10 hours). Less than half of the participants reported insufficient indoor physical activity (209, 39.1% and 138, 37.8%) or insufficient outdoor physical activity (183, 34.2% and 120, 32.9%).

The daily median screen time on all media was 98.6 (60-65.5) minutes, and the screen time on tablets was 41.1 (0-107.2) minutes. The mean total score and score for each domain of the ASQ for the total sample and tablet sample were 269.3 (SD 33.33) and 269.89 (SD 30.75); 55.05 (SD 7.47) and 54.86 (SD 5.51) for communication); 51.54 (SD 10.39) and 51.61 (SD 10.12) for gross motor; 50.38 (SD 11.26) and 50.45 (SD 10.86) for fine motor; 56.61 (SD 6.92) and 56.82 (SD 6.01) for problem-solving; and 55.73 (SD 7.11) and 56.14 (SD 6.45) for personal-social, respectively. The participant characteristics and total ASQ and domain scores did not significantly differ between the 2 sample populations (*P*>.05).

**Table 1:** Demographic characteristics.

Variables		Total sample	Tablet use sample	*P* value	
**Age, n (%)**					.80
	Approximately 34 months 16 days to 50 months 30 days	54 (10.1)	32 (8.8)		
	Approximately 51 months 0 days to 56 months 30 days	105 (19.6)	74 (20.3)		
	Approximately 57 months 0 days to 66 months 0 days	376 (70.3)	259 (70.9)		
**Gender, n (%)**					.89
	Girl	237 (44.3)	160 (43.8)		
	Boy	298 (55.7)	205 (56.2)		
**Caregiver, n (%)**					.95
	Mother only	297 (55.5)	203 (55.6)		
	Father only	35 (6.5)	22 (6.0)		
	Grand parents	203 (38.0)	140 (38.4)		
**Mother’s education, n (%)**					.98
	≤Secondary school	93 (17.4)	65 (17.8)		
	Senior high school	152 (28.4)	104 (28.5)		
	≥Bachelor	290 (54.2)	196 (53.7)		
**Father’s education, n (%)**					.89
	≤Secondary school	69 (12.9)	51 (13.9)		
	Senior high school	180 (33.6)	120 (32.9)		
	≥Bachelor	286 (53.5)	194 (53.2)		
**Monthly income (US $)^a^, n (%)**					.73
	<1000	145 (27.1)	102 (27.9)		
	Approximately 1000 to 1750	81 (15.1)	61 (16.7)		
	>1750	309 (57.8)	202 (55.4)		
**Single child, n (%)**					.96
	One	272 (50.8)	185 (50.7)		
	Two or more	263 (49.2)	180 (49.3)		
**Day sleep time, n (%)**					.85
	Normal	249 (46.6)	164 (44.9)		
	Insufficient	50 (9.3)	33 (9.1)		
	Excessive	236 (44.1)	168 (46.0)		
**Night sleep time, n (%)**					.92
	Sufficient	123 (23.0)	85 (23.3)		
	Insufficient	412 (77.0)	280 (76.7)		
**Indoor physical activity, n (%)**					.70
	Sufficient	326 (60.9)	227 (62.2)		
	Insufficient	209 (39.1)	138 (37.8)		
**Outdoor physical activity, n (%)**					.68
	Sufficient	352 (65.8)	245 (67.1)		
	Insufficient	183 (34.2)	120 (32.9)		
**Screen guard, n (%)**					.31
	Strictly	159 (29.7)	97 (26.6)		
	Occasionally	376 (70.3)	268 (73.4)		
**Daily total screen time, n (%)**					.69
	<1 hour	103 (19.3)	62 (17.0)		
	1~2 hours	200 (37.4)	139 (38.1)		
	>2 hours	232 (43.3)	164 (44.9)		
**Score of ASQ^b^, Mean (SD)**					
	Total score of ASQ	269.3 (33.33)	269.89 (30.75)	.79	
	Score of Communication	55.05 (7.47)	54.86 (5.51)	.71	
	Score of gross motor	51.54 (10.39)	51.61 (10.12)	.92	
	Score of fine motor	50.38 (11.26)	50.45 (10.86)	.93	
	Score of problem-solving	56.61 (6.92)	56.82 (6.01)	.62	
	Score of personal-social	55.73 (7.11)	56.14 (6.45)	.37	

^a^Monthly income: The original options are <7500 RMB, 7500-12500 RMB, >12500 RMB. To convert the amounts from RMB to US dollars, multiply them by 0.137.

^b^ASQ: Ages and Stages Questionnaire.

### Associations Between Screen Exposure Type and ASQ-3 Scores

The univariate associations between demographic characteristics and lifestyle variables and ASQ-3 scores are shown in Table S2 in Multimedia Appendix 1. Age, gender, maternal and paternal educational attainment, and monthly income were associated with the total ASQ score and domain scores (*P*<.05). The results of the univariate association between screen exposure types and ASQ-3 scores are shown in Table S3 in Multimedia Appendix 1. Smartphone screen time, iPad screen time, education content category, animated or realistic content, co-viewing programs, and co-playing games were associated with the total ASQ score and the domain scores (*P*<.05).

Furthermore, as shown in Table S4 in Multimedia Appendix 1, the results of the fully adjusted linear regression model suggested that older age, compared with an age of approximately 34 months 16 days to 50 months 30 days, was more strongly associated with the personal-social score (β=2.29; 95% CI 0.02-4.56; *P*=.048 and β=2.79; 95% CI 0.75-4.83; *P*=.008). Male gender was negatively associated with the total ASQ score (β=–8.92; 95% CI –14.41 to –3.43; *P*=.002), fine motor score (β=–4.16, 95% CI –6.04 to –2.28; *P*<.001), and personal-social score (β=–2.52; 95% CI –3.71 to –1.33; *P*<.001). Comparing with no exposure to smartphones, the total ASQ-3 score, gross motor scores were negatively associated with the use of smartphones for less than 30 minutes (β=–8.62; 95% CI –15.58 to –1.67; *P*=.01 and β=–3.16, 95% CI –5.4 to –0.92; *P*=.01), from 30-60 minutes (β=–13.28; 95% CI –21.98 to –4.58, *P*=.003 and β=–4.52; 95% CI –7.32 to –1.71; *P*=.002) and for more than 60 minutes (β=–12.89; 95% CI –24.75 to –1.02; *P*=.03 and β=–4.71; 95% CI –8.54 to –0.88; *P*=.02). The duration of iPad screen time exceeding 1 hour (β=2.45; 95% CI 0.39-4.51; *P*=.02) had a more negative association with the fine motor score than did never using iPads. Engagement in co-playing games with caregivers was positively associated with the total ASQ-3 score (β=11.64; 95% CI 2.66-20.62; *P*=.01), communication (β=2.19; 95% CI 0.1-4.27; *P*=.04), and fine motor scores (β=4.76; 95% CI 1.68-7.84; *P*=.003), respectively.

### Associations Between Screen Content and ASQ-3 Score

The Spearman correlation coefficients between the screen content and ASQ-3 scores based on the HSVC data are shown in Table S5 in Multimedia Appendix 1. The results showed that education content, pace, screen interaction, animated and realistic content, date, and screen time duration were associated with the total ASQ-3 score, fine motor score, problem-solving score, and personal-social score (*P*<.05).

Furthermore, the results of the median regression between screen content and the ASQ-3 score after adjusting for age, gender, monthly income, co-viewing programs, and co-playing games are shown in Table S6 in Multimedia Appendix 1. This study showed that the median total score on the ASQ-3 was negatively associated with screen time for noneducational content (β=–.055; 95% CI –0.148 to –0.006; *P*=.03), educational and noneducational content (β=–.042, 95% CI –0.081 to –0.007; *P*=.001), and fast-paced content (β=–.034; 95% CI –0.062 to –0.011; *P*=.049). The median gross motor score was negatively associated with screen time for PG-13 rated content (β=–.015; 95%CI –0.022 to 0.009; *P*=.03), educational and noneducational content (β=–.018, 95% CI –0.038 to –0.001; *P*=.02), static content (β=–.022, 95% CI –0.050 to 0.007; *P*=.02). This study also found that the median fine motor score was negatively associated with the time spent viewing G rated content (β=–.032; 95% CI –0.057 to –0.003; *P*=.006), PG rated content (β=–.020; 95% CI –0.036 to –0.007; *P*=.004), noneducational content (β=–.026; 95% CI –0.067 to –0.003; *P*=.01), both educational and noneducational content (β=–.020; 95% CI –0.034 to –0.0010; *P*<.001), fast-paced content (β=–.022; 95% CI –0.033 to –0.014; *P*<.001), static content (β=–.034; 95% CI –0.050 to 0.018; *P*<.001), animated content (β=–.038; 95% CI –0.069 to –0.001; *P*=.004), and screen use during the daytime (β=–.026; 95% CI –0.043 to 0.005; *P*=.005). This study suggested that screen time during the daytime and the time spent viewing touch-based content were negatively associated with the median problem-solving (β=–.017; 95% CI –0.050 to –0.007; *P*=.03) and personal-social scores (β=–.016; 95% CI –0.033 to 0.003; *P*=.006), respectively.

## Discussion

### Principal Findings

Based on 7 days of monitoring data collected by the HSVC application from 2 representative samples of kindergarten children aged 34.5 to 66 months, we analyzed the association of different screen content types and the ASQ-3 score. We found that greater exposure to noneducational programs, fast-paced content, static, animated content, and screens during the daytime was associated with a lower median gross motor score and median fine motor score after controlling for covariates; moreover, greater exposure to noneducational programs and fast speed programs was also associated with lower total ASQ-3 scores.

The results showed that the mean (SD) total screen time and tablet screen time was 111.6 (140.9) minutes and 31.1 (42.5) minutes per day for children aged 34 months 16 days to 50 months 30 days, 172.7 (286.5) minutes and 41.2 (45.5) minutes per day for children aged 51 months 0 days to 56 months 30 days, and 159.3 (191.5) minutes and 42.4 (52.3) minutes per day for children aged 57 months 0 days to 66 months 0 days; these findings are similar to previous studies [32-34]. Whether educational programs are beneficial for early development in toddlers and preschool children is still controversial. This study also did not observe a positive or negative association; however, a significant finding was revealed that exposure to noneducational content was negatively associated with early development (total score on the ASQ-3) or fine motor in preschool children included in this monitoring study. Although there is little direct evidence of a relationship between the ASQ score and its domain scores, several longitudinal studies have shown that exposure to noneducational content before 3 years of age is negatively related to executive functioning and cognitive development at older ages, which are fundamental to the development of fine motor [35-37]. Another longitudinal study confirmed that screen use time was negatively correlated with fine motor skills 1 year after birth among children aged 3-6 years by cross-lagged path analysis [38]. The earlier cross-lagged conclusion may be explained by the results of this study, in which screen time in relation to categories containing both educational and noneducational content was still negatively associated with fine motor skills because it is inevitable to avoid noneducational content for preschoolers.

This study also revealed that exposure to fast-paced content (more than 6 times per minute) was negatively associated with the total ASQ score and fine motor score, while these relationships were not found for low-paced (less than 2 times per minute) or medium-paced (2 to 6 times per minute) content. Furthermore, the daily tablet screen time via tablet for fast-paced content (76.4 minutes) was far greater than that for medium- (8 minutes) and slow-paced content (9.1 minutes); namely, the predominant pace of the screen content for children aged 2-6 years was fast, which is consistent with the findings of previous studies showing that preschool children often view fast-paced content, with rapid screenshots and angle changes [18]. Several controlled experimental studies have shown that watching fast-paced television significantly impairs executive function immediately in children aged 4-6 years [17,18], and these test programs could appropriately reflect fine motor, problem-solving, communication, and personal-social skills.

Touch-enabled media technological devices (tablets and smartphones) play an important role not only because of their growing popularity among children but also because of their influence on early development; the use of tablets and smartphones was associated with decreased scores on the ASQ and its domains in this study, while other media did not show this relationship. Although exposure to touch-enabled content was not associated with the ASQ score, exposure to static content was negatively associated with gross motor and fine motor scores. There is some experimental evidence that toddlers can more readily learn from touchscreen devices than from televisions [39], possibly because the use of tablets is beneficial for motor development in preschoolers [40].

Animated content is widespread in children’s media, and even realistic programs incorporate unrealistic elements. This study revealed that exposure to fantastic and both fantastic and realistic content was negatively associated with the fine motor score; these results are consistent with previous experimental studies [17]. In the working memory system, the content of screen stimuli is encoded, processed, and stored, while animated content is difficult to process because there is no stored schematic to use for interpreting animated content, especially for children’s immature brains [17,41]. Engagement in such activities could lead to a decrease in local neurotransmitters in the lateral prefrontal cortex, subsequently resulting in a reduced availability of these neurotransmitters [42].

This study also revealed that general audience and PG content were associated with lower fine motor scores, while PG-13 content was associated with lower gross motor scores. A plausible explanation regarding this result may be that regardless of what the suggested audience of the screen content is, screen use can replace the time spent on physical activity [12,43]. A previous study revealed that the viewing of PG-13 and restricted content was associated with higher hyperactivity and aggression scores and a lower social skills rating. However, this association was not found in early childhood [44].

### Limitations and Strengths

To our knowledge, this study was the first to examine preschool children’s tablet screen time for different content types via 7-day intelligent monitoring records. By analyzing a sample of representative kindergarteners, we analyzed the associations between screen time and exposure to content types and early development. Several limitations of this study, however, should be mentioned.

First, because of the 7-day monitoring design, we could not calculate the causal relationship between screen exposure and early development. Longitudinal prospective studies and randomized clinical trials are needed to determine causal and cause-and-effect relationships regarding genetic and environmental factors. Second, this monitoring study could accurately measure tablet screen exposure, while other media, such as televisions and smartphones, were measured via questionnaires on the frequency of different types of screen use; thus, the HSVC application should be appropriately used in other devices, such as smartphones or game machines. Third, although the participants were from developed provinces in China, we recruited kindergarteners from urban and suburban areas, which might influence the generalizability of the results to lower-income districts because all areas in Shanghai have relatively high incomes compared with the national average income level. Fourth, in the screen time assessment collected by questionnaires, a limitation was that no distinction was made between weekends and weekdays in the screen time question asked to families and the real-time screen time calculation, which may result in the existence of deviations in the estimation of the screen time. Fifth, although we informed parents to let children use tablets according to their usual habits at home, this process might still increase or change children’s screen exposure behaviors to some extent. In future studies, we will explore ways to make parents accept the installation of intelligent monitoring technology on their own tablets and smartphones so as to restore children’s screen exposure behaviors to the greatest extent possible. Finally, although this survey investigated the age intervals of children according to the age versions of ASQ-3, this study failed to investigate the specific dates of birth of children. Therefore, it may affect the rigor of statistical analysis to some extent.

### Conclusions

This monitoring study showed that screen time associated with exposure to different content types was significantly associated with early development among preschool children, especially for noneducational, static, fast-paced, and animated content, which were associated not only with the total ASQ score but with gross motor and fine motor scores. The highest priority should be placed on limiting the aggregated screen time of noneducational, static, fast-paced, and animated content programs or games, which provide insight into defining high-quality screen content in guidelines.

## Appendix

Multimedia Appendix 1 Additional tables and figures.

## Abbreviations

ASQ: Ages and Stages Questionnaire
ASQ-3: Ages and Stages Questionnaire, Third Edition
HSVC: Healthy Screen Viewing for Children
PG: parental guidance

## Data Availability

The data that support the findings of this study are available on request from the corresponding author upon reasonable request.
