# Supplementary material for: Association of Screen Content With Early Development Among Preschoolers in Shanghai: 7-Day Monitoring Study With Auto Intelligent Technology
Source: J Med Internet Res. 2025 Mar 5;27:e65343. doi: 10.2196/65343 (PMC11928067; doi:10.2196/65343)
Supplement: Multimedia Appendix 1 [file jmir_v27i1e65343_app1.docx]

**Procedures for data collection by IMT**

FigureS1: Overarching framework of the screen data collection workflow.

Note:

1. Tablets of the same model (Huawei MatePad 10.4) are used as the equipment for data acquisition.

b (1). The front-end HSVC app, displaying the usage duration of various applications.

b (2). The screenshot capture feature of the HSVC app requires parental authorization to be enabled.

c (1). The screen usage data is stored on the Huawei Cloud server.

c (2). Researchers download the screenshots through the Huawei Research Platform


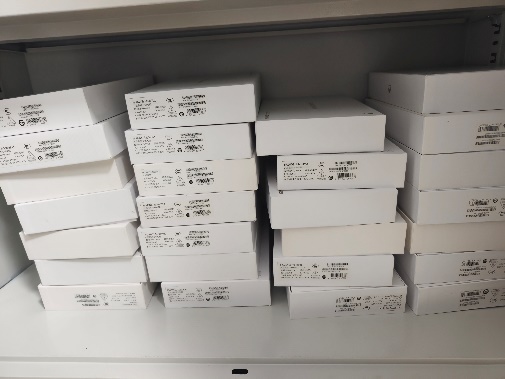

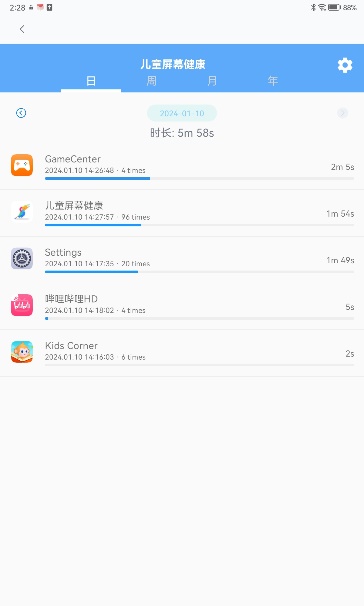

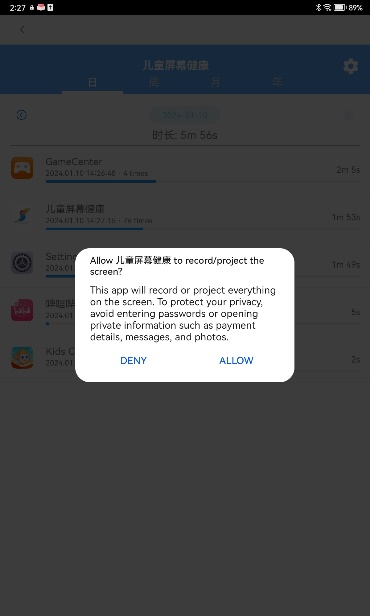


a. b (1). b (2).


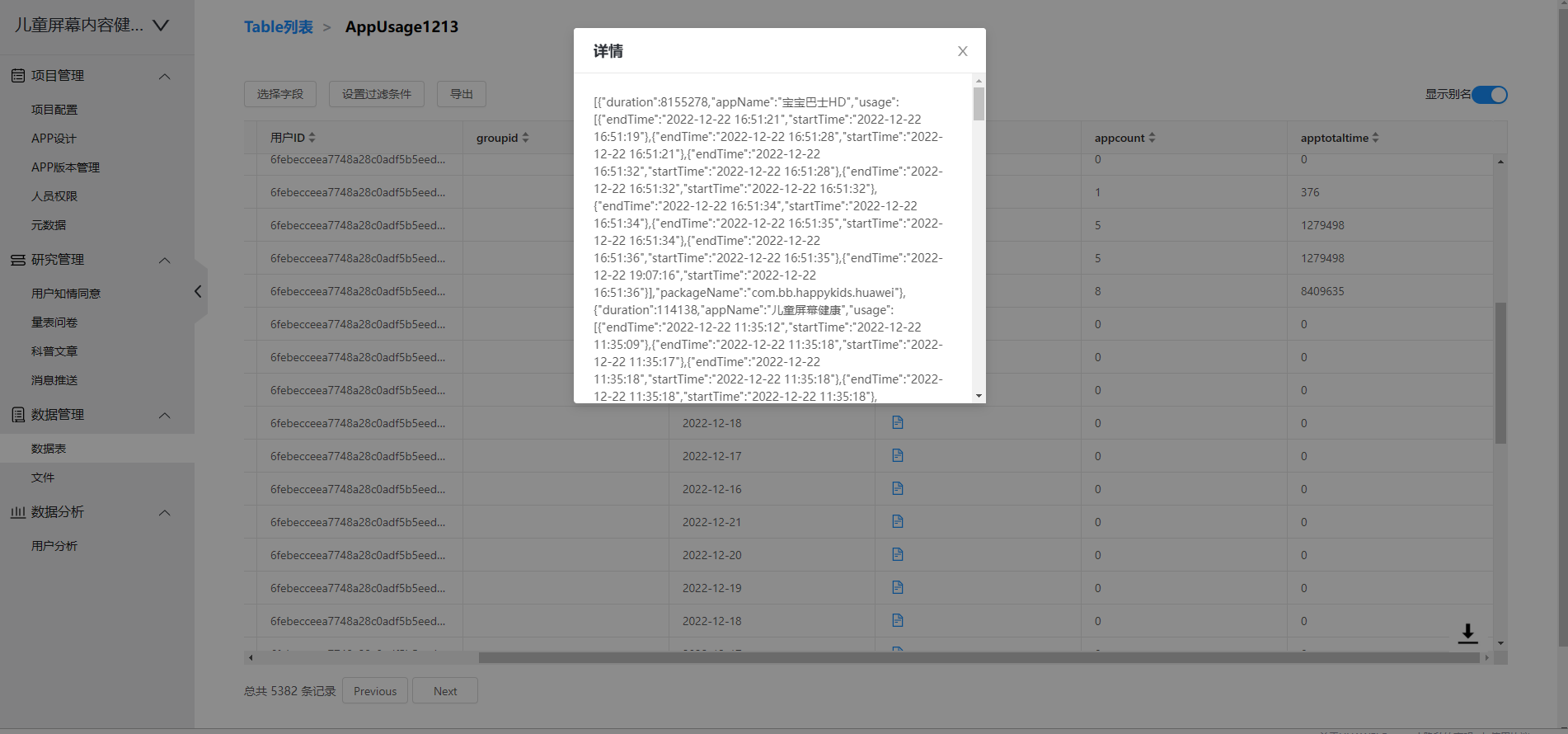
.
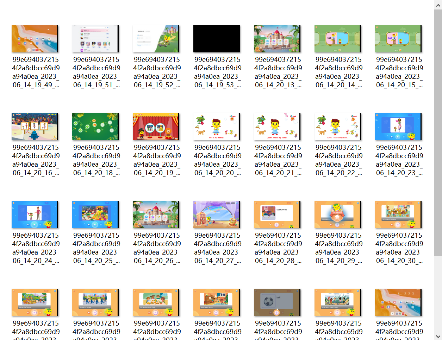


c (1) c (2)

**Classification of type on screen content**

For screen shots, we found their original programs and evaluate the types of screen content, especially for screen paced, we had calculated the average times of scene completely switching/changing speed from 3 times samplings.

**TableS1. Specific standards of six dimensions of screen variables.**

| **Six Dimensions of Screen Variables** | | **Description** |
| --- | --- | --- |
| App Category | Entertainment video | Apps mainly target entertainment video (eg: Netflix, Tencent Video) |
|  | Video game | Apps with interactive entertainment games |
|  | Tool apps | Apps for neither educational nor entertainment, but for specific function, eg, Settings, Camera, Appstore, Maps, Weather. |
|  | Educational | Apps targeted to teach young children knowledge, general knowledge, and skills |
|  | Short video | Apps that focus on distributing short videos (eg, Tiktok, Kwai, Watermelon Video) |
|  |  |  |
| Age restriction | G | Screen contents that all ages admitted; Games suitable for children ＜8. |
|  | PG | Screen content is largely free of sex, horror and violence; Games suitable for children ＜13. |
|  | PG-13 | screen content unsuitable for children＜13, containing violence, nudity, sensuality, and profanity that is not considered serious; Games suitable for children＜17 |
|  | R | Under 17 requires accompanying parent or adult guardian, containing more scenes of sex, violence, drug use, etc. and profanity. |
|  |  |  |
| Educational Contents | Educational Contents | Screen content that aims to educate children in terms of knowledge, common sense, and skills. (e.g., phonics lessons, earthquake safety, Go) |
|  | Non-educational Contents | Screen content that is intended for leisure and entertainment and lacks an overt educational function for children's knowledge, common sense, and skills. (e.g., entertaining cartoons, recreational games) |
|  | Both of them | Screen content whose principal objective is not child education but whose viewing has the potential to foster children's early developmental capabilities like cognition and language. (e.g., nursery rhyme) |
|  |  |  |
| Screen paced | Fast | The scene completely switching/changing over than 6 times during one minute on average |
|  | Medium | The scene completely switching/changing between 2 to 6 times during one minute on average |
|  | Low | The scene completely switching/changing lower than 2 times during one minute on average |
|  |  |  |
| Screen Interaction | Static | Screen content that young children only need to passively view. (eg, programs, movies, short videos) |
|  | Touch-enabled | Screen content that requires young children to actively interact with the screen by tapping, swiping, etc. (eg, video games, chess) |
|  | Caregiver-child interaction | Screen content that requires young children and their caregiver together to actively interact with the screen by tapping, swiping, etc. |
|  |  |  |
| Animated or realistic | Animated Contents | Screenshots correspond to screen content that reflects the virtual world (eg, Tom and Jerry, Peppa Pig) |
|  | Realistic Contents | Screenshots correspond to screen content that reflects the real world (eg, reality show) |
|  | Both of them | Such as feature dramas, Lego stop-motion animation, etc. |

**Table S2. The distribution of ASQ and five domains among demographic characteristic and lifestyles**

|  | N (%) | **ASQ Total score** | | **Communication** | | **Gross motor** | | **Fine motor** | | **Problem-solving** | | **Personal-social** | |
| --- | --- | --- | --- | --- | --- | --- | --- | --- | --- | --- | --- | --- | --- |
|  |  | M (SD) | *P*-value | M (SD) | *P*-value | M (SD) | *P*-value | M (SD) | *P*-value | M (SD) | *P*-value | M (SD) | *P*-value |
| **Age (years)** |  |  | .34 |  | .55 |  | .86 |  | .06 |  | .69 |  | .02 |
| 34 months 16 days ~ 50 months 30 days | 54 (10.1) | 265.56(33.70) |  | 54.54(8.54) |  | 51.39(11.18) |  | 49.91(10.30) |  | 56.11(7.38) |  | 53.61(8.38) |  |
| 51 months 0 days ~ 56 months 30 days | 105 (19.6) | 268.48(26.77) |  | 56.24(6.04) |  | 51.43(9.80) |  | 47.76(11.18) |  | 57.52(5.24) |  | 55.52(6.20) |  |
| 57 months 0 days ~ 66 months 0 days | 376 (70.3) | 270.09(34.93) |  | 54.80(7.65) |  | 51.60(10.46) |  | 51.18(11.32) |  | 56.42(7.25) |  | 56.09(7.12) |  |
| **Gender** |  |  | .01 |  | .58 |  | .62 |  | <.001 |  | .62 |  | <.001 |
| Girl | 237 (44.3) | 273.23 (29.01) |  | 55.25 (7.24) |  | 51.79 (10.08) |  | 52.47 (9.53) |  | 56.77 (6.19) |  | 56.94 (6.16) |  |
| Boy | 298 (55.7) | 266.21 (36.15) |  | 54.90 (7.65) |  | 51.34 (10.64) |  | 48.72 (12.22) |  | 56.48 (7.45) |  | 54.77 (7.16) |  |
| **Caregiver** |  |  | .96 |  | .61 |  | .27 |  | .40 |  | .74 |  | .77 |
| Mother only | 297 (55.5) | 269.26 (34.12) |  | 55.27 (7.21) |  | 50.86 (10.74) |  | 50.74 (11.10) |  | 56.55 (7.03) |  | 55.84 (7.27) |  |
| Father only | 35 (6.5) | 271.29 (29.79) |  | 53.86 (6.98) |  | 55.86 (8.27) |  | 50.29 (11.31) |  | 56.14 (6.07) |  | 55.14 (6.12) |  |
| Grand parents | 203 (38.0) | 269.06 (32.89) |  | 54.95 (7.93) |  | 51.80 (10.04) |  | 49.88 (11.50) |  | 56.77 (6.92) |  | 55.67 (7.07) |  |
| **Education of mother** |  |  | <.001 |  | <.001 |  | <.001 |  | <.001 |  | <.001 |  | .12 |
| ≤Secondary school | 93 (17.4) | 256.51 (36.87) |  | 52.47 (8.33) |  | 48.98 (11.27) |  | 46.94 (12.47) |  | 53.98 (9.54) |  | 54.14 (7.96) |  |
| Senior high school | 152 (28.4) | 268.62 (29.66) |  | 55.36 (6.23) |  | 50.30 (10.33) |  | 49.61 (11.17) |  | 56.97 (5.09) |  | 56.38 (6.49) |  |
| ≥Bachelor | 290 (54.2) | 273.79 (32.98) |  | 55.72 (7.62) |  | 53.02 (9.91) |  | 51.90 (10.63) |  | 57.26 (6.58) |  | 55.90 (7.09) |  |
| **Education of father** |  |  | <.001 |  | .001 |  | .001 |  | <.001 |  | .002 |  | .30 |
| ≤Secondary school | 69 (12.9) | 259.49 (30.29) |  | 53.33 (7.56) |  | 50.14 (9.93) |  | 46.23 (12.11) |  | 54.86 (6.80) |  | 54.93 (6.72) |  |
| Senior high school | 180 (33.6) | 264.67 (35.74) |  | 54.22 (7.78) |  | 49.56 (11.44) |  | 49.22 (12.34) |  | 56.00 (7.61) |  | 55.67 (7.29) |  |
| ≥Bachelor | 286 (53.5) | 274.62 (31.54) |  | 56.00 (7.13) |  | 53.13 (9.55) |  | 52.12 (9.94) |  | 57.41 (6.38) |  | 55.96 (7.10) |  |
| **Monthly income** |  |  | <.001 |  | <.001 |  | <.001 |  | <.001 |  | <.001 |  | <.001 |
| <7500 | 145 (27.1) | 257.45 (30.29) |  | 52.83 (8.82) |  | 49.10 (11.43) |  | 47.34 (13.49) |  | 54.07 (10.05) |  | 54.10 (8.18) |  |
| 7500~12500 | 81 (15.1) | 264.32 (35.74) |  | 54.88 (7.11) |  | 49.44 (10.64) |  | 48.83 (11.13) |  | 56.17 (5.26) |  | 55.00 (7.87) |  |
| >12500 | 309 (57.8) | 276.20 (31.54) |  | 56.15 (6.61) |  | 53.24 (9.48) |  | 52.22 (9.70) |  | 57.91 (4.88) |  | 56.68 (6.17) |  |
| **Single Child** |  |  | .29 |  | .07 |  | .71 |  | .20 |  | .10 |  | .98 |
| One | 272 (50.8) | 270.83 (31.20) |  | 55.63 (7.16) |  | 51.38 (10.23) |  | 50.99 (11.00) |  | 57.10 (6.34) |  | 55.74 (6.44) |  |
| Two or more | 263 (49.2) | 267.76 (35.40) |  | 54.47 (7.75) |  | 51.71 (10.57) |  | 49.75 (11.50) |  | 56.10 (7.45) |  | 55.72 (7.76) |  |
| **Disease** |  |  | .22 |  | .99 |  | .45 |  | .049 |  | .97 |  | .12 |
| None | 57 (10.7) | 269.21 (33.95) |  | 54.74 (7.53) |  | 52.11 (10.39) |  | 50.44 (10.87) |  | 55.79 (7.72) |  | 56.14 (6.75) |  |
| 1~2 | 162 (30.3) | 273.36 (28.32) |  | 55.28 (7.12) |  | 51.91 (10.09) |  | 52.50 (9.86) |  | 57.16 (5.46) |  | 56.52 (6.11) |  |
| >2 | 613 (59.0) | 267.26 (35.44) |  | 55.00 (7.65) |  | 51.25 (10.56) |  | 49.29 (11.85) |  | 56.47 (7.42) |  | 55.25 (7.62) |  |
| **Screen guard** |  |  | .29 |  | .43 |  | .58 |  | .23 |  | .83 |  | .10 |
| Strictly | 159 (29.7) | 271.54 (30.58) |  | 55.44 (7.30) |  | 51.92 (9.84) |  | 51.23 (10.06) |  | 56.51 (7.04) |  | 56.45 (6.15) |  |
| Occasionally | 376 (70.3) | 268.38 (34.43) |  | 54.89 (7.54) |  | 51.38 (10.62) |  | 50.03 (11.72) |  | 56.65 (6.88) |  | 55.42 (7.47) |  |
| **Day sleep time** |  |  | .09 |  | .53 |  | .11 |  | .05 |  | .67 |  | .11 |
| Normal | 249 (46.6) | 272.59 (30.33) |  | 55.38 (7.07) |  | 52.47 (9.65) |  | 51.36 (10.52) |  | 56.87 (6.19) |  | 56.51 (6.40) |  |
| Insufficient | 50 (9.3) | 262.00 (48.94) |  | 53.90 (10.07) |  | 49.60 (13.16) |  | 50.20 (11.82) |  | 55.30 (10.12) |  | 53.0 (10.45) |  |
| Excessive | 236 (44.1) | 267.42 (32.07) |  | 54.96 (7.25) |  | 50.97 (10.43) |  | 49.39 (11.83) |  | 56.61 (6.82) |  | 55.49 (6.83) |  |
| **Night sleep time** |  |  | .96 |  | .66 |  | .32 |  | .87 |  | .60 |  | .59 |
| Sufficient | 123 (23.0) | 269.43 (28.67) |  | 54.80 (7.42) |  | 52.32 (9.44) |  | 50.53 (10.83) |  | 56.34 (6.27) |  | 55.45 (6.49) |  |
| Insufficient | 412 (77.0) | 269.28 (34.64) |  | 55.13 (7.49) |  | 51.31 (10.66) |  | 50.34 (11.39) |  | 56.69 (7.11) |  | 55.81 (7.29) |  |
| **Indoor physical activity** |  |  | .78 |  | .74 |  | .52 |  | .20 |  | .66 |  | .69 |
| Sufficient | 326 (60.9) | 268.99 (32.49) |  | 54.97 (7.42) |  | 51.78 (10.16) |  | 49.89 (11.54) |  | 56.72 (6.57) |  | 55.63 (6.94) |  |
| Insufficient | 209 (39.1) | 269.83 (34.69) |  | 55.19 (7.56) |  | 51.17 (10.76) |  | 51.15 (10.78) |  | 56.44 (7.44) |  | 55.89 (7.39) |  |
| **Outdoor physical activity** |  |  | .90 |  | .81 |  | .63 |  | .78 |  | .27 |  | .94 |
| Sufficient | 352 (65.8) | 269.18 (32.49) |  | 55.11 (7.21) |  | 51.70 (10.00) |  | 50.28 (11.14) |  | 56.36 (6.75) |  | 55.71 (6.74) |  |
| Insufficient | 183 (34.2) | 269.59 (34.69) |  | 54.95 (7.97) |  | 51.23 (11.13) |  | 50.57 (11.51) |  | 57.08 (7.23) |  | 55.77 (7.81) |  |
| **Daily total screen time** |  |  | .88 |  | .33 |  | .73 |  | .49 |  | .89 |  | .99 |
| < 1 hour | 103 (19.3) | 268.84 (38.35) |  | 54.37 (8.79) |  | 51.60 (10.76) |  | 50.68 (11.27) |  | 56.31 (8.49) |  | 55.87 (7.76) |  |
| 1~2 hours | 200 (37.4) | 270.25 (32.87) |  | 55.13 (7.37) |  | 51.80 (10.65) |  | 50.75 (10.86) |  | 57.00 (5.76) |  | 55.58 (7.44) |  |
| >2 hours | 232 (43.3) | 268.73 (31.42) |  | 55.30 (6.91) |  | 51.29 (10.04) |  | 49.94 (11.61) |  | 56.40 (7.08) |  | 55.80 (6.60) |  |

**Table S3. The distribution of ASQ and five domains among screen use styles**

|  | N (%) | **ASQ Total score** | | **Communication** | | **Gross motor** | | **Fine motor** | | **Problem-solving** | | **Personal-social** | |
| --- | --- | --- | --- | --- | --- | --- | --- | --- | --- | --- | --- | --- | --- |
|  |  | M (SD) | *P*-value | M (SD) | *P*-value | M (SD) | *P*-value | M (SD) | *P*-value | M (SD) | *P*-value | M (SD) | *P*-value |
| **Age first use** |  |  | .46 |  | .73 |  | .54 |  | .94 |  | .60 |  | .11 |
| <2 years | 127 (23.7) | 265.79 (39.21) |  | 54.33 (7.98) |  | 50.71 (11.16) |  | 49.61 (12.5) |  | 56.02 (8.44) |  | 55.12 (8.42) |  |
| 2~3years | 203 (37.9) | 271.55 (30.82) |  | 55.74 (6.98) |  | 52.02 (9.83) |  | 51.28 (10.58) |  | 57.02 (6.26) |  | 55.49 (7.09) |  |
| >3 years | 205 (38.3) | 269.29 (31.71) |  | 54.83 (7.6) |  | 51.59 (10.46) |  | 49.98 (11.09) |  | 56.56 (6.49) |  | 56.34 (6.19) |  |
| **TV screen time** |  |  | .36 |  | .74 |  | .71 |  | .08 |  | .88 |  | .43 |
| 0 | 110 (20.6) | 269.45 (38.82) |  | 54.55 (8.87) |  | 51.36 (11.02) |  | 51.77 (11.18) |  | 56.05 (8.62) |  | 55.73 (7.72) |  |
| 1-30 | 154 (28.8) | 270.52 (36.97) |  | 55.65 (7.43) |  | 52.05 (10.86) |  | 50.36 (12.2) |  | 56.56 (6.97) |  | 55.91 (7.46) |  |
| 30-60 | 158 (29.5) | 271.39 (24.23) |  | 55.35 (6.36) |  | 51.52 (9.62) |  | 50.57 (10.13) |  | 57.72 (4.32) |  | 56.23 (5.99) |  |
| >60 | 113 (21.1) | 264.65 (33.32) |  | 54.34 (7.49) |  | 51.06 (10.25) |  | 48.81 (11.44) |  | 55.66 (7.78) |  | 54.78 (7.45) |  |
| **PC screen time** |  |  | .44 |  | .71 |  | .40 |  | .57 |  | .33 |  | .91 |
| 0 | 306 (57.2) | 270.42 (32.71) |  | 55.33 (7.44) |  | 51.81 (10.12) |  | 50.64 (10.48) |  | 56.96 (7.08) |  | 55.69 (6.98) |  |
| 1-30 | 117 (21.9) | 267.74 (37.77) |  | 54.19 (7.88) |  | 51.2 (10.94) |  | 49.96 (12.65) |  | 56.2 (6.91) |  | 56.2 (7.65) |  |
| 30-60 | 73 (13.6) | 268.22 (30.32) |  | 55.27 (6.87) |  | 51.92 (10.43) |  | 50.48 (11.58) |  | 55.55 (7) |  | 55 (7.36) |  |
| >60 | 39 (7.3) | 267.44 (30.11) |  | 55.13 (7.65) |  | 49.74 (10.94) |  | 49.49 (12.4) |  | 57.05 (5.35) |  | 56.03 (6.09) |  |
| **Cell phone screen time** |  |  | <.001 |  | .003 |  | <.001 |  | .01 |  | .01 |  | .02 |
| 0 | 138 (25.8) | 277.39 (22.94) |  | 56.45 (5.48) |  | 54.02 (7.61) |  | 52.25 (8.82) |  | 57.83 (4.35) |  | 56.85 (5.89) |  |
| 1-30 | 247 (46.2) | 268.99 (35.83) |  | 55.2 (7.41) |  | 51.4 (10.94) |  | 50.12 (12.01) |  | 56.52 (7.84) |  | 55.75 (6.94) |  |
| 30-60 | 108 (20.2) | 262.73 (37.66) |  | 53.19 (9.18) |  | 49.63 (11.81) |  | 49.86 (11.53) |  | 55.51 (7.26) |  | 54.54 (8.72) |  |
| >60 | 42 (7.9) | 261.67 (30.22) |  | 54.4 (7.82) |  | 49.17 (9.69) |  | 47.14 (12.4) |  | 55.95 (6.74) |  | 55 (6.9) |  |
| **PAD screen time** |  |  | .04 |  | .39 |  | .11 |  | .16 |  | .009 |  | .13 |
| 0 | 87 (16.3) | 264.14 (37.44) |  | 55.23 (6.9) |  | 49.83 (11.22) |  | 49.25 (11.75) |  | 55.17 (9.07) |  | 54.66 (8.2) |  |
| 1-30 | 206 (38.5) | 268.57 (36.73) |  | 54.76 (8.05) |  | 51.43 (10.8) |  | 50.19 (12.07) |  | 56.43 (7.57) |  | 55.75 (7.44) |  |
| 30-60 | 140 (26.2) | 270.25 (30.74) |  | 54.57 (7.83) |  | 52.36 (9.68) |  | 50.5 (10.82) |  | 56.89 (5.8) |  | 55.93 (6.69) |  |
| >60 | 102 (19.1) | 273.97 (24.39) |  | 56.18 (6.06) |  | 52.11 (9.71) |  | 51.57 (9.65) |  | 57.79 (4.14) |  | 56.32 (5.88) |  |
| **Screen content** |  |  | .23 |  | .43 |  | .28 |  | .46 |  | .78 |  | .07 |
| G | 148 (27.7) | 268.14 (39.6) |  | 55.44 (7.8) |  | 51.25 (11.75) |  | 49.86 (12.05) |  | 56.42 (8.67) |  | 55.17 (8.15) |  |
| PG | 289 (54) | 268.15 (32.45) |  | 54.67 (7.5) |  | 51.18 (10.18) |  | 50.22 (11.14) |  | 56.56 (6.41) |  | 55.52 (7.28) |  |
| R | 98 (18.3) | 274.54 (24.08) |  | 55.61 (6.86) |  | 53.06 (8.63) |  | 51.63 (10.32) |  | 57.04 (5.22) |  | 57.19 (4.19) |  |
| **Education** |  |  | .17 |  | .64 |  | .29 |  | .046 |  | .32 |  | .11 |
| Education | 178 (33.3) | 271.49 (30.23) |  | 55.03 (7.3) |  | 52.08 (10.01) |  | 51.38 (9.95) |  | 56.91 (6.53) |  | 56.1 (6.45) |  |
| Entertainment | 200 (37.4) | 265.78 (36.18) |  | 55.4 (7.02) |  | 50.62 (10.87) |  | 48.83 (12.34) |  | 56.02 (7.41) |  | 54.9 (8.31) |  |
| Both | 157 (29.3) | 271.37 (32.72) |  | 54.65 (8.21) |  | 52.1 (10.18) |  | 51.24 (11.05) |  | 57.01 (6.7) |  | 56.37 (6.03) |  |
| **Fantastical or realistic** |  |  | <.001 |  | <.001 |  | .005 |  | <.001 |  | .005 |  | .04 |
| Fantastical | 333 (62.2) | 264.86 (35.72) |  | 54.19 (7.99) |  | 50.68 (10.81) |  | 48.93 (11.96) |  | 55.87 (7.8) |  | 55.2 (7.58) |  |
| Realistic | 68 (12.7) | 281.47 (23.72) |  | 57.94 (4.25) |  | 55.15 (8.51) |  | 54.19 (8.79) |  | 58.38 (4.18) |  | 55.81 (6.61) |  |
| Both | 134 (25) | 274.22 (29.04) |  | 55.75 (7) |  | 51.87 (9.84) |  | 52.05 (9.85) |  | 57.54 (5.27) |  | 57.01 (5.95) |  |
| **Paced** |  |  | .62 |  | .86 |  | .94 |  | .26 |  | .17 |  | .67 |
| Fast | 133 (24.9) | 268.46 (33.86) |  | 55.19 (7.14) |  | 51.54 (10.75) |  | 50.49 (10.53) |  | 55.75 (8.11) |  | 55.49 (7.3) |  |
| Low | 204 (38.1) | 268.09 (32.91) |  | 54.83 (7.52) |  | 51.72 (10.28) |  | 49.44 (11.37) |  | 56.57 (6.82) |  | 55.54 (7.24) |  |
| Medium | 198 (37) | 271.16 (33.5) |  | 55.2 (7.67) |  | 51.36 (10.31) |  | 51.29 (11.58) |  | 57.22 (6.08) |  | 56.09 (6.87) |  |
| **Interactive** |  |  | .31 |  | .70 |  | .40 |  | .92 |  | .06 |  | .08 |
| Parent-child | 153 (28.6) | 268.3 (31.99) |  | 54.8 (7.5) |  | 51.67 (10.2) |  | 50.2 (11.25) |  | 56.01 (7.66) |  | 55.62 (6.57) |  |
| Child only | 139 (26) | 272.41 (29.31) |  | 55.58 (6.84) |  | 51.4 (10.34) |  | 50.86 (11.11) |  | 57.7 (4.56) |  | 56.87 (5.56) |  |
| Non | 166 (31) | 270.24 (34.49) |  | 55.15 (7.97) |  | 52.32 (10.23) |  | 50.42 (10.96) |  | 56.84 (6.84) |  | 55.51 (7.56) |  |
| Both | 77 (14.4) | 263.77 (39.57) |  | 54.42 (7.48) |  | 49.87 (11.18) |  | 49.81 (12.29) |  | 55.32 (8.67) |  | 54.35 (9.19) |  |
| **Coviewing program** |  |  | <.001 |  | <.001 |  | <.001 |  | .03 |  | <.001 |  | <.001 |
| seldom | 214 (40) | 261.29 (40.6) |  | 53.71 (8.78) |  | 49.49 (11.61) |  | 49.09 (12.66) |  | 54.95 (9.38) |  | 54.04 (8.85) |  |
| sometimes | 136 (25.4) | 270.18 (28) |  | 54.85 (7.04) |  | 52.43 (9.68) |  | 50.07 (9.77) |  | 56.58 (5.19) |  | 56.25 (6.23) |  |
| often | 185 (34.6) | 277.97 (24.31) |  | 56.76 (5.59) |  | 53.27 (8.95) |  | 52.11 (10.34) |  | 58.54 (3.22) |  | 57.3 (4.66) |  |
| **Coplaying game** |  |  | <.001 |  | <.001 |  | <.001 |  | <.001 |  | <.001 |  | <.001 |
| seldom | 195 (36.4) | 259.87 (40.47) |  | 53.82 (8.5) |  | 49.23 (11.58) |  | 48.1 (13.01) |  | 54.82 (9.41) |  | 53.9 (9.01) |  |
| sometimes | 164 (30.7) | 270.24 (28.88) |  | 54.36 (7.35) |  | 52.35 (9.81) |  | 50.27 (10.08) |  | 56.98 (4.94) |  | 56.28 (6.38) |  |
| often | 176 (32.9) | 278.92 (24.59) |  | 57.07 (5.79) |  | 53.35 (9.01) |  | 53.01 (9.56) |  | 58.24 (4.35) |  | 57.24 (4.5) |  |

Table S4. Linear regression analysis for ASQ and each domain on demographic and screen exposure styles

|  | **ASQ Total score** | | **Communication** | | **Gross motor** | | **Fine motor** | | **Problem-solving** | | **Personal-social** | |  |
| --- | --- | --- | --- | --- | --- | --- | --- | --- | --- | --- | --- | --- | --- |
|  | β (95%CI) | *P*-value | β (95%CI) | *P*-value | β (95%CI) | *P*-value | β (95%CI) | *P*-value | β (95%CI) | *P*-value | β (95%CI) | *P*-value |  |
| **Age (years)** |  |  |  |  |  |  |  |  |  |  |  |  |  |
| 34 months 16 days ~ 50 months 30 days | 1 (ref.) |  | 1 (ref.) |  | 1 (ref.) |  | 1 (ref.) |  | 1 (ref.) |  | 1 (ref.) |  |  |
| 51 months 0 days ~ 56 months 30 days | 2.46 (-8.04, 12.95) | .65 | 0.88 (-1.56, 3.31) | .48 | -0.11 (-3.5, 3.27) | .95 | -1.87 (-5.47, 1.72) | .31 | 1.27 (-0.94, 3.48) | .26 | 2.29 (0.02, 4.56) | .048 |  |
| 57 months 0 days ~ 66 months 0 days | 3.43 (-6.01, 12.87) | .48 | -0.29 (-2.48, 1.89) | .79 | -0.12 (-3.17, 2.92) | .94 | 1.05 (-2.19, 4.28) | .53 | 0.01 (-1.98, 2) | .99 | 2.79 (0.75, 4.83) | .008 |  |
| **Sex** |  |  |  |  |  |  |  |  |  |  |  |  |  |
| Girl | 1 (ref.) |  | 1 (ref.) |  | 1 (ref.) |  | 1 (ref.) |  | 1 (ref.) |  | 1 (ref.) |  |  |
| Boy | -8.92 (-14.41, -3.43) | .002 | -0.77 (-2.04, 0.51) | .24 | -0.96 (-2.73, 0.82) | .29 | -4.16 (-6.04, -2.28) | <.001 | -0.52 (-1.68, 0.64) | .38 | -2.52 (-3.71, -1.33) | <.001 |  |
| **Education of mother** |  |  |  |  |  |  |  |  |  |  |  |  |  |
| ≤Secondary school | 1 (ref.) |  | 1 (ref.) |  | 1 (ref.) |  | 1 (ref.) |  | 1 (ref.) |  | 1 (ref.) |  |  |
| Senior high school | 3.52 (-6.64, 13.68) | .50 | 1.42 (-0.93, 3.77) | .24 | -0.13 (-3.4, 3.15) | .94 | -0.36 (-3.84, 3.12) | .84 | 1.42 (-0.72, 3.56) | .19 | 1.17 (-1.03, 3.37) | .30 |  |
| ≥Bachelor | 3.05 (-8.55, 14.65) | .61 | 0.63 (-2.05, 3.32) | .64 | 1.03 (-2.71, 4.77) | .59 | -0.06 (-4.03, 3.92) | .98 | 0.92 (-1.53, 3.36) | .46 | 0.52 (-1.99, 3.03) | .69 |  |
| **Education of father** |  |  |  |  |  |  |  |  |  |  |  |  |  |
| ≤Secondary school | 1 (ref.) |  | 1 (ref.) |  | 1 (ref.) |  | 1 (ref.) |  | 1 (ref.) |  | 1 (ref.) |  |  |
| Senior high school | -1.19 (-11.88, 9.49) | .83 | -0.58 (-3.05, 1.9) | .65 | -2 (-5.45, 1.45) | .26 | 1.81 (-1.86, 5.47) | .33 | 0.01 (-2.25, 2.26) | .99 | -0.43 (-2.74, 1.88) | .72 |  |
| ≥Bachelor | 1.03 (-11.36, 13.41) | .87 | 0.17 (-2.7, 3.04) | .91 | -0.57 (-4.57, 3.42) | .78 | 2.82 (-1.42, 7.07) | .19 | -0.1 (-2.71, 2.51) | .94 | -1.3 (-3.98, 1.38) | .34 |  |
| **Monthly income** |  |  |  |  |  |  |  |  |  |  |  |  |  |
| <7500 | 1 (ref.) |  | 1 (ref.) |  | 1 (ref.) |  | 1 (ref.) |  | 1 (ref.) |  | 1 (ref.) |  |  |
| 7500~12500 | 2.62 (-6.2, 11.45) | .56 | 1.32 (-0.72, 3.37) | .20 | -0.85 (-3.7, 1.99) | .56 | 0.7 (-2.33, 3.72) | .65 | 0.99 (-0.87, 2.85) | .30 | 0.47 (-1.44, 2.38) | .63 |  |
| >12500 | 13.81 (6.43, 21.19) | <.001 | 2.17 (0.46, 3.88) | .01 | 2.56 (0.18, 4.94) | .04 | 3.42 (0.89, 5.95) | .008 | 2.92 (1.36, 4.47) | <.001 | 2.74 (1.14, 4.33) | .001 |  |
| **TV screen time** |  |  |  |  |  |  |  |  |  |  |  |  |  |
| 0 | 1 (ref.) |  | 1 (ref.) |  | 1 (ref.) |  | 1 (ref.) |  | 1 (ref.) |  | 1 (ref.) |  |  |
| 1-30 | 2.8 (-5.07, 10.67) | .49 | 1.75 (-0.08, 3.57) | .06 | 1.17 (-1.36, 3.71) | .36 | -0.57 (-3.26, 2.13) | .68 | 0.5 (-1.16, 2.16) | .56 | -0.06 (-1.76, 1.65) | .95 |  |
| 30-60 | 3.99 (-4.05, 12.03) | .33 | 1.52 (-0.34, 3.38) | .11 | 0.52 (-2.07, 3.11) | .69 | -0.43 (-3.19, 2.32) | .76 | 1.88 (0.19, 3.57) | .03 | 0.51 (-1.23, 2.25) | .57 |  |
| >60 | 1.55 (-7.17, 10.26) | .73 | 0.66 (-1.36, 2.68) | .52 | 1.74 (-1.07, 4.55) | .23 | -0.94 (-3.92, 2.04) | .54 | 0.41 (-1.42, 2.25) | .66 | -0.33 (-2.21, 1.56) | .73 |  |
| **PC screen time** |  |  |  |  |  |  |  |  |  |  |  |  |  |
| 0 | 1 (ref.) |  | 1 (ref.) |  | 1 (ref.) |  | 1 (ref.) |  | 1 (ref.) |  | 1 (ref.) |  |  |
| 1-30 | 0.63 (-6.49, 7.74) | .86 | -0.42 (-2.07, 1.23) | .62 | 0.48 (-1.81, 2.78) | .68 | -0.05 (-2.49, 2.38) | .97 | 0.01 (-1.49, 1.51) | .99 | 0.61 (-0.93, 2.15) | .44 |  |
| 30-60 | 2.24 (-6.62, 11.1) | .62 | 1.48 (-0.58, 3.53) | .16 | 1.29 (-1.56, 4.15) | .37 | 0.93 (-2.11, 3.96) | .55 | -0.95 (-2.82, 0.91) | .32 | -0.5 (-2.42, 1.41) | .61 |  |
| >60 | -4.32 (-15.94, 7.3) | .47 | 0.02 (-2.67, 2.71) | .99 | -1.81 (-5.56, 1.94) | .34 | -1.86 (-5.84, 2.12) | .36 | 0.09 (-2.35, 2.54) | .94 | -0.77 (-3.28, 1.75) | .55 |  |
| **Cell phone screen time** |  |  |  |  |  |  |  |  |  |  |  |  |  |
| 0 | 1 (ref.) |  | 1 (ref.) |  | 1 (ref.) |  | 1 (ref.) |  | 1 (ref.) |  | 1 (ref.) |  |  |
| 1-30 | -8.62 (-15.58, -1.67) | .02 | -0.79 (-2.4, 0.82) | .34 | -3.16 (-5.4, -0.92) | .006 | -2.4 (-4.79, -0.02) | .048 | -0.97 (-2.43, 0.5) | .19 | -1.3 (-2.81, 0.2) | .09 |  |
| 30-60 | -13.28 (-21.98, -4.58) | .003 | -2.35 (-4.37, -0.34) | .02 | -4.52 (-7.32, -1.71) | .002 | -2.13 (-5.11, 0.85) | .16 | -1.69 (-3.53, 0.14) | .07 | -2.59 (-4.47, -0.7) | .007 |  |
| >60 | -12.89 (-24.75, -1.02) | .03 | -1.28 (-4.03, 1.46) | .36 | -4.71 (-8.54, -0.88) | .02 | -4.11 (-8.18, -0.05) | .047 | -1.21 (-3.71, 1.29) | .34 | -1.57 (-4.13, 1) | .23 |  |
| **PAD screen time** |  |  |  |  |  |  |  |  |  |  |  |  |  |
| 0 | 1 (ref.) |  | 1 (ref.) |  | 1 (ref.) |  | 1 (ref.) |  | 1 (ref.) |  | 1 (ref.) |  |  |
| 1-30 | 2.08 (-6.31, 10.48) | .63 | -0.6 (-2.54, 1.35) | .55 | 1.81 (-0.9, 4.52) | .19 | -0.47 (-3.35, 2.41) | .75 | 0.91 (-0.86, 2.68) | .31 | 0.44 (-1.38, 2.25) | .64 |  |
| 30-60 | 4.62 (-4.57, 13.8) | .32 | -0.68 (-2.81, 1.45) | .53 | 3.18 (0.22, 6.14) | .04 | -0.4 (-3.55, 2.74) | .80 | 1.53 (-0.4, 3.46) | .12 | 0.99 (-0.99, 2.98) | .33 |  |
| >60 | 9.51 (-0.25, 19.28) | .06 | 1.1 (-1.16, 3.37) | .34 | 2.66 (-0.49, 5.81) | .10 | 1.6 (-1.74, 4.95) | .35 | 2.45 (0.39, 4.51) | .02 | 1.69 (-0.42, 3.8) | .12 |  |
| **Screen content** |  |  |  |  |  |  |  |  |  |  |  |  |  |
| G | 1 (ref.) |  | 1 (ref.) |  | 1 (ref.) |  | 1 (ref.) |  | 1 (ref.) |  | 1 (ref.) |  |  |
| PG | 4.53 (-2.12, 11.18) | .18 | 0.22 (-1.33, 1.76) | .78 | 1.14 (-1, 3.29) | .30 | 1.77 (-0.51, 4.05) | .13 | 0.73 (-0.67, 2.13) | .31 | 0.66 (-0.78, 2.1) | .37 |  |
| R | 8.74 (0.32, 17.16) | .04 | 0.44 (-1.51, 2.39) | .66 | 2.79 (0.08, 5.51) | .04 | 2.43 (-0.45, 5.31) | .10 | 0.79 (-0.98, 2.56) | .38 | 2.28 (0.46, 4.1) | .01 |  |
| **Educational** |  |  |  |  |  |  |  |  |  |  |  |  |  |
| Educational | 1 (ref.) |  | 1 (ref.) |  | 1 (ref.) |  | 1 (ref.) |  | 1 (ref.) |  | 1 (ref.) |  |  |
| Entertaining | -3.73 (-10.68, 3.22) | .29 | 0.87 (-0.74, 2.48) | .29 | -0.86 (-3.1, 1.38) | .45 | -2.12 (-4.5, 0.26) | .08 | -0.63 (-2.09, 0.84) | .40 | -0.99 (-2.49, 0.52) | .20 |  |
| Both | -0.59 (-7.86, 6.68) | .87 | -0.54 (-2.22, 1.15) | .53 | 0.76 (-1.58, 3.11) | .52 | -0.71 (-3.2, 1.78) | .58 | -0.13 (-1.67, 1.4) | .86 | 0.03 (-1.55, 1.6) | .98 |  |
| **Fantastical** |  |  |  |  |  |  |  |  |  |  |  |  |  |
| Fantastical | 1 (ref.) |  | 1 (ref.) |  | 1 (ref.) |  | 1 (ref.) |  | 1 (ref.) |  | 1 (ref.) |  |  |
| Realistic | 9.97 (1.16, 18.78) | .03 | 2.99 (0.95, 5.03) | .004 | 3.06 (0.22, 5.9) | .04 | 3.38 (0.37, 6.4) | .03 | 1.42 (-0.43, 3.28) | .13 | -0.89 (-2.8, 1.01) | .36 |  |
| Both | -3.47 (-11.08, 4.14) | .37 | 1.51 (-0.08, 3.09) | .06 | 0.25 (-1.96, 2.46) | .83 | 1.66 (-0.69, 4) | .17 | 1.15 (-0.29, 2.6) | .12 | 0.77 (-0.71, 2.25) | .31 |  |
| **Speed** |  |  |  |  |  |  |  |  |  |  |  |  |  |
| Fast speed | 1 (ref.) |  | 1 (ref.) |  | 1 (ref.) |  | 1 (ref.) |  | 1 (ref.) |  | 1 (ref.) |  |  |
| Low speed | -6.77 (-14.02, 0.47) | .07 | -1.46 (-3.14, 0.21) | .09 | -0.99 (-3.33, 1.35) | .41 | -2.91 (-5.39, -0.42) | .02 | -0.28 (-1.81, 1.24) | .72 | -1.13 (-2.7, 0.43) | .16 |  |
| Medium speed | -5.53 (-14.71, 3.65) | .24 | -0.86 (-2.62, 0.91) | .34 | -1.35 (-3.8, 1.11) | .28 | -0.97 (-3.58, 1.64) | .47 | 0.38 (-1.22, 1.99) | .64 | -0.68 (-2.33, 0.97) | .42 |  |
| **Interactive** |  |  |  |  |  |  |  |  |  |  |  |  |  |
| Parent-child | 1 (ref.) |  | 1 (ref.) |  | 1 (ref.) |  | 1 (ref.) |  | 1 (ref.) |  | 1 (ref.) |  |  |
| Child only | 3.67 (-3.73, 11.08) | .33 | 0.63 (-1.09, 2.35) | .47 | -0.53 (-2.92, 1.86) | .66 | 0.95 (-1.58, 3.49) | .46 | 1.52 (-0.04, 3.08) | .06 | 1.1 (-0.5, 2.71) | .18 |  |
| Non | 1.37 (-6.21, 8.94) | .72 | -0.17 (-1.92, 1.59) | .85 | 0.41 (-2.03, 2.86) | .74 | 0.53 (-2.07, 3.12) | .69 | 0.87 (-0.73, 2.46) | .29 | -0.27 (-1.91, 1.36) | .74 |  |
| Both | -5.53 (-14.71, 3.65) | .24 | -0.4 (-2.52, 1.73) | .71 | -1.95 (-4.91, 1.01) | .20 | -0.8 (-3.94, 2.35) | .62 | -0.85 (-2.78, 1.09) | .39 | -1.54 (-3.53, 0.45) | .13 |  |
| **Coviewing programs** |  |  |  |  |  |  |  |  |  |  |  |  |  |
| Seldom | 1 (ref.) |  | 1 (ref.) |  | 1 (ref.) |  | 1 (ref.) |  | 1 (ref.) |  | 1 (ref.) |  |  |
| Sometimes | 3.28 (-5.02, 11.59) | .44 | 0.61 (-1.32, 2.53) | .54 | 1.61 (-1.07, 4.28) | .24 | -0.84 (-3.69, 2) | .56 | 0.63 (-1.12, 2.38) | .48 | 1.28 (-0.51, 3.08) | .16 |  |
| Often | 4.79 (-3.97, 13.55) | .28 | 0.83 (-1.2, 2.86) | .42 | 1.58 (-1.25, 4.4) | .27 | -1.14 (-4.15, 1.86) | .46 | 1.84 (-0.01, 3.68) | .05 | 1.68 (-0.21, 3.58) | .08 |  |
| **Coplaying games** |  |  |  |  |  |  |  |  |  |  |  |  |  |
| Seldom | 1 (ref.) |  | 1 (ref.) |  | 1 (ref.) |  | 1 (ref.) |  | 1 (ref.) |  | 1 (ref.) |  |  |
| Sometimes | 7.16 (-0.88, 15.21) | .08 | 0.1 (-1.76, 1.97) | .91 | 1.87 (-0.72, 4.47) | .16 | 2.39 (-0.37, 5.14) | .09 | 1.31 (-0.39, 3) | .13 | 1.49 (-0.25, 3.23) | .09 |  |
| Often | 11.64 (2.66, 20.62) | .01 | 2.19 (0.1, 4.27) | .04 | 2.07 (-0.83, 4.97) | .16 | 4.76 (1.68, 7.84) | .003 | 1.08 (-0.81, 2.97) | .26 | 1.54 (-0.4, 3.49) | .12 |  |

Table S5. Spearman correlation between ASQ and types of screen content

| **Screen content** | **Screen time (min)**  [P_50_ (P_50_, P_75_)] | **ASQ Total score** | | **Communication** | | **Gross motor** | | **Fine motor** | | **Problem-solving** | | **Personal-social** | |
| --- | --- | --- | --- | --- | --- | --- | --- | --- | --- | --- | --- | --- | --- |
|  |  | r | *P-value* | r | *P-value* | r | *P-value* | r | *P-value* | r | *P-value* | r | *P-value* |
| **APP category** |  |  |  |  |  |  |  |  |  |  |  |  |  |
| Entertainment video | 1.4 (0, 55.1) | -0.005 | .93 | 0.038 | .48 | -0.042 | .42 | -0.041 | .43 | 0.031 | .55 | 0.019 | .71 |
| Video game | 0 (0, 4.4) | -0.002 | .98 | 0.013 | .81 | -0.001 | .99 | -0.011 | .83 | 0.010 | .58 | -0.050 | .34 |
| Tool | 7.3 (4.8, 12.4) | -0.027 | .61 | -0.089 | .09 | 0.044 | .41 | -0.062 | .24 | -0.056 | .29 | 0.009 | .86 |
| Educational | 73.6 (11.5, 147.9) | **-0.104** | **.047** | -0.055 | .30 | -0.055 | .30 | **-0.112** | **.03** | -0.040 | .44 | -0.049 | .35 |
| Short video | 0 (0, 13.9) | 0.010 | .85 | 0.069 | .19 | 0.012 | .82 | -0.034 | .52 | -0.024 | .65 | -0.044 | .40 |
| **Age restriction ^a^** |  |  |  |  |  |  |  |  |  |  |  |  |  |
| G | 45.6 (21.7, 45.6) | -0.080 | .13 | 0.012 | .82 | -0.026 | .63 | **-0.155** | **.003** | -0.038 | .47 | -0.042 | .43 |
| PG-13 | 0 (0, 6.2) | -0.037 | .48 | 0.038 | .47 | -0.035 | .50 | -0.045 | .39 | -0.059 | .26 | -0.070 | .18 |
| PG | 2.4 (0, 146.0) | -0.013 | .81 | 0.007 | .89 | 0.021 | .70 | -0.044 | .41 | 0.067 | .21 | 0.026 | .62 |
| **Educational content** |  |  |  |  |  |  |  |  |  |  |  |  |  |
| Educational | 37.1 (0.3, 116.4) | -0.036 | .50 | -0.023 | .63 | -0.023 | .66 | -0.070 | .18 | 0.009 | .86 | 0.020 | .71 |
| Non-educational | 24.1 (9.1, 53.4) | -0.054 | .30 | -0.024 | .65 | 0.018 | .73 | **-0.111** | **.04** | -0.003 | .96 | **-0.105** | **.045** |
| Both | 16.4 (3.3, 84.3) | -0.102 | .05 | 0.003 | .96 | -0.086 | .10 | **-0.119** | **.02** | -0.029 | .58 | -0.039 | .46 |
| **Screen paced** |  |  |  |  |  |  |  |  |  |  |  |  |  |
| Low paced | 9.1 (5.3, 17.4) | -0.012 | .82 | -0.041 | .44 | 0.036 | .49 | -0.056 | .29 | -0.027 | .60 | -0.014 | .79 |
| Medium paced | 8.0 (0, 113.6) | -0.024 | .65 | -0.050 | .34 | 0.004 | .94 | -0.015 | .79 | 0.024 | .62 | -0.098 | .06 |
| Fast paced | 76.4 (22.5, 159.5) | **-0.111** | **.03** | -0.018 | .74 | -0.067 | .21 | **-0.174** | **<.001** | -0.007 | .89 | -0.028 | .60 |
| **Screen interaction*** |  |  |  |  |  |  |  |  |  |  |  |  |  |
| Non-interactive | 41.2 (17.1, 86.8) | -0.102 | .05 | -0.029 | .58 | -0.062 | .24 | **-0.171** | **.001** | -0.028 | .60 | -0.013 | .81 |
| Child-interactive | 21.9 (4.0, 69.4) | -0.048 | .37 | 0.010 | .84 | 0.057 | .28 | -0.034 | .52 | 0.012 | .82 | **-0.108** | **.04** |
| **Fantastical or realistic** |  |  |  |  |  |  |  |  |  |  |  |  |  |
| Fantastical | 41.4 (16.7, 73.6) | -0.094 | .07 | -0.009 | .86 | -0.024 | .64 | **-0.163** | **.002** | 0.024 | .65 | -0.059 | .26 |
| Realistic | 0 (0, 366.3) | 0.027 | .60 | 0.067 | .20 | 0.009 | .86 | -0.009 | .87 | -0.004 | .94 | -0.009 | .86 |
| Both | 1.0 (0, 12.5) | -0.014 | .79 | -0.039 | .46 | 0.003 | .96 | -0.023 | .66 | 0.024 | .65 | 0.081 | .12 |
| **Date** |  |  |  |  |  |  |  |  |  |  |  |  |  |
| Workday | 43.8 (21.1, 81.3) | -0.112 | .03 | -0.068 | .19 | -0.029 | .58 | **-0.170** | **.001** | -0.039 | .46 | -0.058 | .27 |
| Weekend | 36.1 (0, 96.9) | -0.012 | .81 | 0.046 | .38 | 0.034 | .51 | **-0.084** | **.01** | 0.021 | .69 | -0.081 | .12 |
| **Time period** |  |  |  |  |  |  |  |  |  |  |  |  |  |
| Daytime | 26.5 (3.4, 71.1) | **-0.123** | **.02** | -0.089 | .09 | -0.037 | .48 | **-0.154** | **.003** | **-0.118** | **.02** | -0.088 | .09 |
| Nighttime | 47.0 (19.9, 86.9) | -0.070 | .18 | -0.034 | .52 | 0.008 | .88 | **-0.144** | **.006** | 0.040 | .44 | -0.092 | .08 |
| Deep nighttime | 28.0 (0.1, 89.1) | -0.009 | .87 | 0.040 | .44 | 0.010 | .86 | -0.042 | .422 | -0.002 | .98 | -0.028 | .60 |

^a^ For age restriction, there are only less than 1% screen time exposure to R age restriction content and parent-child interaction, thus these content results of R age restriction caregiver-child interaction cannot be calculated.

Table S6. Median regression analysis for ASQ and each domain on demographic and types of screen contents

|  | **ASQ Total score** | | **Communication** | | **Gross motor** | | **Fine motor** | | **Problem-solving** | | **Personal-social** | |
| --- | --- | --- | --- | --- | --- | --- | --- | --- | --- | --- | --- | --- |
|  | β (95%CI) | *P*-value | β (95%CI) | *P*-value | β (95%CI) | *P*-value | β (95%CI) | *P*-value | β (95%CI) | *P*-value | β (95%CI) | *P*-value |
| **Content restriction ^a^** |  |  |  |  |  |  |  |  |  |  |  |  |
| G | -0.001  (-0.120,0.034) | .98 | 0.003  (-0.001,0.013) | .57 | <-0.001  (-0.030,0.025) | .98 | **-0.032**  **(-0.057,-0.003)** | **.006** | -0.006  (-0.030,0.008) | .20 | -0.002  (-0.020,0.007) | .71 |
| PG-13 | -0.004  (-0.015,0.035) | .81 | <0.001  (-0.001,0.007) | .94 | **-0.015**  **(-0.022,0.009)** | **.03** | <-0.001  (-0.022,0.007) | .97 | 0.003(-0.007,0.006) | .29 | 0.001  (-0.004,0.006) | .82 |
| PG | -0.035  (-0.058,0.002) | .08 | -0.003  (-0.014,0.007) | .34 | <-0.001  (-0.010,0.005) | .99 | **-0.020**  **(-0.036,-0.007)** | **.004** | -0.004  (-0.018,0.001) | .12 | -0.005  (-0.010,-0.002) | .06 |
| **Educational content** |  |  |  |  |  |  |  |  |  |  |  |  |
| Educational | 0.004  (-0.014,0.028) | .67 | <0.001  (<-0.001,<0.001) | .72 | 0.006  (-0.008,0.014) | .30 | -0.001  (-0.016,0.005) | .87 | -0.001  (-0.012,0.008) | .85 | 0.002  (<0.001,0.005) | .27 |
| Non-educational | **-0.055**  **(-0.148,-0.006)** | **.03** | <-0.001  (-0.002,<-0.001) | .90 | -0.010  (-0.036,0.023) | .51 | **-0.026**  **(-0.067,-0.003)** | **.01** | -0.003  (-0.021,0.023) | .69 | -0.007  (-0.025,-0.004) | .06 |
| Both | **-0.042**  **(-0.081,-0.007)** | **.001** | <0.001  (<0.001,<0.001) | .68 | **-0.018**  **(-0.038,<-0.001)** | **.02** | **-0.020**  **(-0.034,-0.010)** | **<.001** | -0.003  (-0.020,0.005) | .50 | -0.004  (-0.015,0.002) | .05 |
| **Screen paced** |  |  |  |  |  |  |  |  |  |  |  |  |
| Low speed | -0.007  (-0.172,0.264) | .95 | <-0.001  (<-0.001, <-0.001) | .48 | 0.025  (-0.055,0.052) | .55 | 0.012  (-0.041,0.055) | .68 | -0.052  (-0.244,-0.001) | .13 | 0.050  (-0.097,0.106) | .42 |
| Medium speed | -0.007  (-0.039,0.024) | .71 | <0.001  (<0.001, <0.001) | .96 | 0.004  (-0.012,0.015) | .54 | 0.001  (-0.004,0.013) | .89 | 0.001  (-0.021,0.004) | .92 | -0.015  (-0.035,-0.001) | .18 |
| Fast speed | **-0.034**  **(-0.062,-0.011)** | **.049** | <-0.001  (<-0.001, <-0.001) | .60 | -0.009  (-0.020,0.005) | .21 | **-0.022**  **(-0.033,-0.014)** | **<.001** | 0.001  (-0.013,0.015) | .83 | -0.005  (<-0.001,0.011) | .65 |
| **Screen interaction*** |  |  |  |  |  |  |  |  |  |  |  |  |
| Non-interactive | -0.046  (-0.086,0.002) | .08 | <0.001  (<-0.001, <0.001) | .89 | **-0.022**  **(-0.050,0.007)** | **.02** | **-0.034**  **(-0.050,-0.018)** | **<.001** | -0.005  (-0.053,0.023) | .81 | <-0.001  (-0.012,0.007) | .91 |
| Child interactive | 0.019  (-0.048,0.060) | .52 | <-0.001  (<-0.001, <0.001) | .87 | 0.015  (-0.004,0.037) | .13 | 0.002  (-0.021,0.013) | .80 | -0.024  (-0.049,0.030) | .34 | **-0.016**  **(-0.033,0.003)** | **.006** |
| **Fantastical or realistic** |  |  |  |  |  |  |  |  |  |  |  |  |
| Fantastical | -0.009  (-0.116,0.034) | .81 | 0.002  (-0.001,0.012) | .73 | <-0.001  (-0.031,0.014) | .99 | **-0.038**  **(-0.069,-0.001)** | **.004** | 0.014  (-0.054, <-0.001) | .37 | -0.009  (-0.027, -0.001) | .13 |
| Realistic | 0.014  (-0.009,0.043) | .44 | 0.003  (<-0.001,0.008) | .39 | -0.004  (-0.021,0.009) | .56 | 0.002  (-0.008,0.008) | .74 | 0.009  (-0.011,0.019) | .25 | 0.003  (0.001, 0.006) | .33 |
| Both | -0.035  (-0.059,-0.013) | .10 | -0.003  (-0.014,0.004) | .45 | <-0.001  (-0.009,0.006) | .99 | -0.013  (-0.033,-0.002) | .08 | -0.001  (-0.022,0.006) | .94 | -0.004  (-0.010,-0.001) | .17 |
| **Date** |  |  |  |  |  |  |  |  |  |  |  |  |
| Workday | 0.006  (-0.096,0.041) | .85 | -0.007  (-0.017,0.011) | .27 | -0.017  (-0.033,<-0.001) | .14 | -0.017  (-0.042,0.005) | .19 | -0.006  (-0.026,0.012) | .83 | 0.006  (-0.014,0.026) | .51 |
| Weekend | -0.038  (-0.084,0.029) | .07 | 0.007  (-0.001,0.012) | .09 | 0.011  (-0.019,0.018) | .14 | -0.014  (-0.028,0.004) | .08 | -0.013  (-0.030,-0.003) | .43 | -0.008  (-0.022,0.003) | .16 |
| **Time period** |  |  |  |  |  |  |  |  |  |  |  |  |
| Daytime | -0.013  (-0.057,0.015) | .73 | -0.005  (-0.018,-0.001) | .35 | -0.002  (-0.035,0.009) | .89 | **-0.026**  **(-0.043,0.005)** | **.005** | **-0.017**  **(-0.050, -0.007)** | **.03** | 0.009  (-0.043,0.034) | .65 |
| Nighttime | 0.010  (-0.060,0.067) | .81 | 0.003  (-0.007,0.008) | .62 | 0.008  (-0.007,0.025) | .58 | -0.004  (-0.034,0.015) | .68 | 0.014  (0.008, 0.038) | .08 | -0.025  (-0.054,0.031) | .23 |
| Deep nighttime | -0.011  (-0.061,0.021) | .61 | 0.004  (<-0.001,0.0088) | .17 | -0.007  (-0.017,0.012) | .37 | -0.008  (-0.015,-0.001) | .15 | 0.001  (-0.014,0.008) | .86 | 0.002  (-0.027,0.013) | .83 |

^a^ For age restriction, there are only less than 1% screen time exposure to R age restriction content and parent-child interaction, thus these content results of R age restriction caregiver-child interaction cannot be calculated.
